# Demonstration of Cases of Nervous Disease

**Published:** 1910-11-26

**Authors:** Purves Stewart


### Hospital Clinics.

### DEMONSTRATION OF CASES OF NERVOUS DISEASE.

By PURVES STEWART, M.D., F.R.C.P.

(At the Polyclinic, Chenies Street, W.C., on Tuesday, October 18, 1910.)

Gentlemen,—I show you first a case of unusual occupation-neurosis. You are, of course, familiar with such neuroses as writer's cramp, telegraphist's cramp, piano-player's palsy; but here is a man with barber's cramp. He tells us that when he shaves a man he has a shivering and shaking of the hand. This, as you will readily understand, is embarrassing for the person shaved. Notice that it is only shaving which brings out this defect; when he makes wigs, for instance, an action which requires equally fine movements, the hairs of the wig having to be sewn in, his hand does not tremble. But he gets cramp in the back of his shoulders and in the back of his arm when he shaves. These occupation-neuroses are due not to fatigue of muscles, but to fatigue of certain centres in the brain, which preside over certain groups of movements. A man who has got writer's cramp may be a good tennis player or billiard player, and may even be able to squeeze dumb-bells to the maximum amount. It is of no use these people wasting their time trying to develop their muscles, because they are quite well developed. They must stop the particular skilled action altogether and rest their brain; they need not rest their limb. This man may go into some other occupation and not feel it. We usually cut off the skilled action for three months. In this case, after that time we shall advise him to use a safety razor, which will be a somewhat different kind of action for his brain-cells.

### Two Illustrative Cases.

I now want to show you two interesting and contrasting cases. Here is a boy of twenty, a clerk, whose history is as follows: Two years ago he was sitting at his desk writing, when he suddenly lost power in the right hand, dropped his pen, and could not move the hand at all. The whole limb became weak in a few minutes. He was feverish for a week, and found that he could not move his fingers, his wrist, or his elbow—in fact, he was paralysed from the elbow downwards. He had no pain or loss of feeling, and except for the fever and the weakness of the right arm he was well. In other words, he had a typical attack of acute anterior poliomyelitis, and his limb subsequently wasted. I ask you to notice the peculiar distribution of the muscular atrophy in this boy's hand; it is rather unusual. His pupils are equal and normal, and they react to light. The optic discs and cranial nerves are normal, so are his sensory functions; he has no cutaneous anæsthesia or analgesia. All his limbs are powerful, with the exception of the right upper limb, and that is the only part where the reflexes are not normal. He has, as you see, a triceps jerk. You see he has slight wasting of the right upper arm muscles, which, however, are not completely paralysed. Pronation and supination are good. The movements of the wrist are all right, but all the intrinsic muscles of his hand are atrophied. He has got claw-hand with atrophy of the interossei, of the lumbricales and intrinsic muscles of the thumb. The thenar and hypothenar muscles are good. You see he has got the *main en griffe*. He cannot extend his inter-phalangeal joints unless they are fixed for him. I want you to notice particularly that his pupils are equal and normal, because it is in that respect I want you to compare him with the next patient. Except for the difference in age, their clinical conditions, to all appearances, might lead you to imagine they were twin brothers. But the history in the two cases is different.

This second patient is fifty-three years of age, and twelve months ago he was playing with some little children when he fell and dislocated his right shoulder. Next evening it was reduced under an anæsthetic, and he now comes saying that he feels his arm weak ever since the accident, especially below the elbow, and that he cannot use his hand for writing. He has got practically the same distribution of muscular atrophy as the boy had. His right pupil is slightly smaller than the left, his right eye is sunken inwards a little, and the right palpebral fissure is a little narrower than the left. This is the keynote of the differential diagnosis between the two. There are signs of a lesion of a cervical sympathetic on the affected side. Apart from that, his cranial nerves are normal. There is no cutaneous anæsthesia or analgesia, no motor weakness, with the exception of his right hand. Pronation and supination are good. He has total atrophy of the intrinsic muscles of his hand, and he, like the boy, has got typical claw-hand, with hollowing of the interossei on the back of the hand; he has interosseal paralysis with semiflexion of the fingers at the inter-phalangeal joints, and extension of the metacarpo-phalangeal joints.

### Diagnostic Differences.

There is a great difference in the lesion in the two cases. Number one case—the boy—obviously had acute anterior poliomyelitis affecting his lower cervical and upper thoracic segments, but this has cleared up, with the exception of the first thoracic segment, which, as you know, supplies the intrinsic muscles of the hand. In the second patient you will remember he had a dislocation, which was reduced under an anæsthetic. What probably happened was that, not during the dislocation but during the reduction of it, the patient had some root of his brachial plexus torn or stretched. The nerve which is commonly injured in those cases is the lowest trunk of the brachial plexus. The first dorsal nerve can be easily damaged during the reduction of a shoulder-dislocation. What evidence have we that it is a lesion of the first dorsal root and not of the spinal cord? We have evidence that the cervical sympathetic is affected, whose fibres emerge in the first dorsal root and run up the neck to reach the eye. In the boy's case these were not affected at all. So that here we have a lesion of the first thoracic root. This man tells us that at one time he had some numbness along the arm. This condition was originally described by Madame Klumpke, as she was before she married Professor Déjerine. The cervical sympathetic thus gives us a clue as to the lesion in one case being extra-medullary, and in the other intra-medullary. Even if we had not had the histories, what I have shown you would have enabled us to find this out.

Here is a boy with ordinary pseudo-hypertrophic paralysis. He has the usual type of myopathy. There is atrophy of many muscles in the lower ana upper extremities, and false enlargement of other muscles. Notice his waddling gait, and when he lies down flat how it is impossible for him to rise again without helping himself by climbing up his own legs. He has large quadriceps muscles and relatively large calves. He arches his back, and when he walks upstairs he can only do so with great difficulty. Otherwise there is nothing characteristic about him. The pectorals are fairly good. The latissimus dorsi is still present, and his weakness is chiefly confined to the extensors of his hips and knees.

### A Case of Neuritis.

The next case I want to show you was a very deceptive one. He is a soldier, aged twenty-nine, who came to see me three months ago at hospital with a great many of the signs of myopathy. He had to climb up his own legs from the floor in the typical myopathic fashion. The history was interesting. This condition had come on three months before. Previously having been quite well, he first noticed weakness of his legs; then he became feeble in his arms, and noticed that he gradually became unable to walk upstairs, owing to the weakness of the extensors of the hips and knees. His previous history was unimportant, except that he had enteric fever in Africa eight years ago while in the army. There was no history of venereal disease, he was a temperate man in the matter of alcohol, and there was no similar disease in any relative. When we saw him three months ago he also had weakness of the arms, resulting in drop-wrist, as you will see from the photograph, in which he was trying to dorsiflex his wrists, but could not. We found that his pupils and cranial nerves were normal, also the sensory functions. In the upper extremities he had paralysis of the extensors of the wrists and fingers. The intrinsic muscles of the hand were normal. The trunk muscles were all right, but in the lower extremities he had marked weakness of all the muscles of the hips and thighs, and could not lift his legs off the ground against gravity; neither could he walk upstairs. When on the ground he had to climb up his legs in order to get up, exactly like a myopathic patient; he was so like a patient with that condition that I hastily entered him as a case of myopathy, a little later in onset than one usually sees in such cases. The only unusual thing which struck one about him was that he had marked drop-wrist, which is unusual in myopathy. There is one form of myopathy, which has been described within the last year or so, called myotonia atrophica, to which this patient closely corresponds. In this there is weakness of the extensors of the wrists, weakness of the legs, some stiffness of muscles, and often weakness of the sterno-mastoids. This man had no affection of the latter, nor any notable muscular rigidity, but in other respects he corresponded to the description of myotonia atrophica. No case of myotonia atrophica has yet been investigated pathologically, and I therefore got him to come into hospital with the idea of examining the affected muscles. I took, under an anæsthetic, pieces of the supposed myopathic muscles, the calf, the quadriceps, etc., and also pieces out of the healthy trapezius. To our disappointment—though to the patient's satisfaction—the muscles were found to be normal, none of them showing any trace of myopathy or anything abnormal. He had loss of faradic excitability in all the affected muscles, without any polar changes, just as in myopathy. A still more interesting point is that whilst we had him in hospital—a period of three months—he proceeded to improve, and has now recovered the power of extending his wrists; in fact, he is quite well. You will see that he can get up from the floor without aid. The moral to be drawn from this is that one is tempted to look upon some of the conditions known as myotonia atrophica as possibly simply aberrant forms of peripheral neuritis. The chances are that this man has had an abnormal distribution of peripheral neuritis, from which he has got well. I thought it instructive to bring him up and confess the mistake we had made in this man's case. We could trace no cause for the condition, such as lead, arsenic, or alcohol.

### Another Case.

The next man whom you will now see has been sent to this clinique to-day, and I know nothing about him. You shall hear his history from himself. He is sixty years of age, and his work is that of clerk and timekeeper. Twelve months ago he began to get giddy in occasional attacks. So we may call it paroxysmal giddiness. He has not had a serious attack for three months, but had a slight one only yesterday. He has had five attacks altogether. When he has an attack he says he loses the use of his arms and legs and of the body generally, and that he would fall if he did not sit down. He also feels sick, retching a good deal, but does not bring much up. During the attack there is also a pain at the top of his head, he is drowsy, and there is singing in his ears. He also becomes temporarily deaf. The last bad attack lasted two hours, and his hands were cold and clammy, and he perspired profusely. I think we may conclude that his is a fairly typical case of Menière's syndrome, otherwise called labyrinthine vertigo.

This is the kind of case which really should go to the aurist. We can exclude ordinary cerebellar or cerebral disease, because the condition comes on paroxysmally. He is not unconscious in the attack. We should observe whether there are any signs referable to the auditory nerve. We should really have had a syringe to cool down the labyrinth, to see if we could produce the normal vestibular nystagmus or not. In some people with disease of the vestibular nerve you can no longer produce vestibular nystagmus by syringing the ear with cold water. This man has no nystagmus at present. He can hear the watch tick only on contact on both sides, so that he is fairly deaf in both ears. Has he any other signs about him? because such a diagnosis does not take us very far unless we know what is inside the ear.

Various theories have been brought forward with regard to Menière's disease. Menière himself described a case in which he had an opportunity of examining the ear anatomically, where there was a hæmorrhage into the labyrinth; but numerous other cases have since been observed in which there has been no hæmorrhage. The most feasible explanation of Menière's disease which has been offered is that given by Mr. Arthur Cheatle. He regards Menière's disease as the result of a sudden increase or alteration of tension in the endolymph of the membranous labyrinth, just as in a patient with acute glaucoma there is a sudden increase of intraocular tension. If Mr. Cheatle is right we should be able to cure some cases of Menière's syndrome by tapping the labyrinth, and I think that has been done in some cases. There have been cases of operations on one or other semicircular canals in patients with inveterate paroxysmal vertigo, and this is the sort of case where one would like to get Mr. Cheatle to examine the patient to see if the endolymph is under tension. His theory is further supported by the fact that certain cases of Menière's disease are relieved by lumbar puncture. If we take away some cerebrospinal fluid we diminish the intra-cerebral tension, and we know the endolymph is in continuity with the cerebro-spinal fluid in the sub-arachnoid space. If we saw a patient of this sort during an attack it would be worth while to try the effect of lumbar puncture and see what happened. I can recall two cases of inveterate vertigo which were relieved for a long time by a single lumbar puncture. Those other patients had previously run the gauntlet of eminent aurists, so that all the usual things had been done to them beforehand, and it was only as a last resort that we did lumbar puncture.

I think this man should first of all have any abnormal local ear condition put right, and if that does not cure him, the question of lumbar puncture might be well considered. Many of these cases do not require severe measures. Sometimes if we put a strong blister over the mastoid we can alter the interlabyrinthine circulation sufficiently to relieve those attacks. With regard to drugs, I think aural surgeons are in the habit of prescribing hydrobromic acid and very small doses of quinine—one-twelfth of a grain.

